# Viral Response among Early Treated HIV Perinatally Infected Infants: Description of a Cohort in Southern Mozambique

**DOI:** 10.3390/healthcare10112156

**Published:** 2022-10-28

**Authors:** Maria Grazia Lain, Paula Vaz, Marco Sanna, Nalia Ismael, Sérgio Chicumbe, Teresa Beatriz Simione, Anna Cantarutti, Gloria Porcu, Stefano Rinaldi, Lesley de Armas, Vinh Dinh, Suresh Pallikkuth, Rajendra Pahwa, Paolo Palma, Nicola Cotugno, Savita Pahwa

**Affiliations:** 1 Fundação Ariel Glaser Contra o SIDA Pediátrico, Maputo P.O.Box 2822, Mozambique; 2 Research Unit of Clinical Immunology and Vaccinology, Children’s Hospital Bambino Gesù, IRCCS, 0165 Rome, Italy; 3 Technological Platforms Department, Instituto Nacional de Saúde, Marracuene, Maputo 1120, Mozambique; 4 Health System and Policy Program, Instituto Nacional de Saúde, Marracuene, Maputo 1120, Mozambique; 5 National STI, HIV/AIDS Control Program, Maputo P.O. Box 264, Mozambique; 6 National Centre for Healthcare Research and Pharmaco-Epidemiology, Unit of Biostatistics, Epidemiology and Public Health, Department of Statistics and Quantitative Methods, University of Milano-Bicocca, 20126 Milan, Italy; 7 Department of Microbiology and Immunology, University of Miami Miller School of Medicine, Miami, FL 33136, USA; 8 Department of Systems Medicine, University of Rome “Tor Vergata”, 0133 Rome, Italy

**Keywords:** viral load suppression, viral rebound, pediatric HIV, early antiretroviral therapy, drug resistance, adherence

## Abstract

Early initiation of antiretroviral therapy and adherence to achieve viral load suppression (VLS) are crucial for reducing morbidity and mortality of perinatally HIV-infected infants. In this descriptive cohort study of 39 HIV perinatally infected infants, who started treatment at one month of life in Mozambique, we aimed to describe the viral response over 2 years of follow up. VLS ≤ 400 copies/mL, sustained VLS and viral rebound were described using a Kaplan–Meier estimator. Antiretroviral drug transmitted resistance was assessed for a sub-group of non-VLS infants. In total, 61% of infants reached VLS, and 50% had a rebound. Cumulative probability of VLS was 36%, 51%, and 69% at 6, 12 and 24 months of treatment, respectively. The median duration of VLS was 7.4 months (IQR 12.6) and the cumulative probability of rebound at 6 months was 30%. Two infants had resistance biomarkers to drugs included in their treatment regimen. Our findings point to a low rate of VLS and high rate of viral rebound. More frequent viral response monitoring is advisable to identify infants with rebound and offer timely adherence support. It is urgent to tailor the psychosocial support model of care to this specific age group and offer differentiated service delivery to mother–baby pairs.

## 1. Introduction

Remarkable results were achieved in reducing new HIV infections among infants after the Global plan for elimination of pediatric HIV and the Start Free, Stay Free, AIDS Free framework [1,2]. However, in 2020, there were still 150.000 new pediatric infections, the majority living in Sub Saharan Africa and acquiring the infection through vertical transmission, and 99.000 deaths [3].

Early identification and treatment initiation of infants has increased over time in priority countries [4,5,6,7,8]. Starting antiretroviral treatment (ART) as early as possible, to achieve viral suppression is crucial to reduce infants’ morbidity and mortality [9,10,11,12]. In addition, initiating ART in the first 3 months of life has shown better immunologic and virologic responses [12,13].

Viral suppression must be sustained to achieve long-term benefits and allow infants to grow into adolescence and adulthood as well as to prevent HIV-related complications [12,13]. Maintaining undetectable viremia also prevents ART resistance development [14,15,16]. This is a major constraint in Africa, where available drugs for younger patients are limited and the first line regime must be preserved as much as possible [17,18,19].

Viral suppression in children (0–14 years old) is reported at 40% globally and 43% in Southern Africa [3]. Sub-optimal long-term viral response (43–63%) among children in low and middle-income countries (LMIC) compared to children in high-income countries has been described in a meta-analysis [20].

Infants starting ART below the age of 2 months have demonstrated a great variability in achieving viral suppression from 19% to 81% [21], and generally had worse viral responses compared to older children [22]. Existing evidence shows that achieving and sustaining viral control in infants starting ART in the first days of life is difficult [23,24], but little is known about the challenges to sustain viral control and the risk of rebound in infants starting ART in the first month of life.

Adherence correlates with viral suppression in children and infants [25,26,27] and suboptimal adherence was shown to be a major contributor to poor ART response in the pediatric population [28]. In children, especially in infants, adherence to ART and consequently good and sustained virologic response, are related to caregivers adherence behavior [24] and to retention into care, with youngest age being one of the risk factors for attrition [29].

Mozambique has a high HIV burden, with approximately 125.000 children living with HIV, about 79% on ART, an estimated HIV vertical transmission of 12% and around 71% of HIV-exposed infants accessing diagnosis within 2 months of life in 2021 [30]. Although, psychosocial support (PSS) package of care is offered to caregivers integrated into ART care, viral suppression among 0–14 years old children was 51% [30] and programmatic unpublished data suggest that it is worse in infants less than 1 year of age.

There are no data in Mozambique describing viral response in infants who started ART in the first months of life, nor data on sustained viral suppression in this age group. This study aims to describe the viral response among a cohort of infants who started ART at one month of age. Findings will contribute to build knowledge and critical evidence to inform guidelines for improved follow up care for infants and caregivers.

## 2. Materials and Methods

This is a descriptive cohort study of HIV perinatally infected infants diagnosed and recruited within the second month of life in two health centers of Matola district (Machava II, Matola I) and followed at Matola Provincial Hospital, in Maputo province, southern Mozambique. The cohort is part of TARA (Toward AIDS Remission Approaches), funded by the National Institute of Health, NIH (R01AI127347).

The study started in 2017; the cohort included 43 HIV perinatally infected infants: thirty-nine (24 female) had a pre-ART viral load (VL) and at least 3 VL measurements in the first six months after ART initiation, and were included in the analysis of viral response. Four children were excluded from the analysis: 2 had only one visit, with one measurement of the pre-ART VL and then left the study, 1 left the study at 3 months of age and had 3 VL measurements, and 1 died at 2 months of age with one measurement of the pre-ART VL.

Infants started ART with Zidovudine (AZT) or Abacavir (ABC) plus Lamivudine (3TC) and Lopinavir/Ritonavir (LPV/r) and were followed for 2 years with monthly clinical and PSS visits provided by a multidisciplinary team composed of a pediatrician, a nurse and a psychologist. Adherence to treatment was self-reported by the caregiver, similarly to the standard practice in Mozambique [31]. We defined good adherence if VLS was achieved and sustained and poor adherence if VLS was not achieved or was not sustained, in the absence of resistance mutation of ARV drugs used in the child’s regimen.

Samples were collected before starting ART and then at the following intervals: 1, 2, 4, 5, 8, 9, 11, 17, 18, and 23 months after ART initiation. Whole blood was drawn in EDTA tubes and sent to the Molecular Virology and Immunology Laboratory of the Instituto Nacional de Saúde -National Institute of Health (INS) in Maputo. HIV-1 plasma viral load (VL) was quantified using COBAS^®^ AmpliPrep/COBAS^®^ TaqMan^®^ HIV-1 Test, version 2.0 (Roche Diagnostics, Mannheim, Germany) with a limit detection of 20 copies/mL. Reminiscent blood was prepared and stored for peripheral blood mononuclear cell (PBMC) and immunological tests. In case of plasma HIV RNA viral load > 1000 copies/mL, intensive psychosocial support sessions were offered and VL test repeated as per Ministry of Health (MOH) guidelines.

To investigate possible HIV transmitted drug resistance for non-nucleoside reverse transcriptase inhibitor (NNRTI), nucleoside reverse transcriptase inhibitor (NRTIs) and protease inhibitors (PI) from the mother to the child as a possible explanation for the lack of viral suppression, resistance Sanger sequencing of the HIV pol region was performed. Sequencing was done in pre-ART samples from 12/15 of infants who never achieved viral suppression in the 24 months of follow up. HIV-1 RNA was extracted from plasma using the QIAamp Viral RNA Mini Kit according to manufacturer’s instructions. Amplification and sequencing were conducted using the Thermo Fisher Scientific^TM^ HIV-1 Genotyping Kit: Amplification Module Cycle and Genotyping Kit in the Applied Biosystem Genetic Analyzer 3130. The sequences were generated using ReCall and drug resistance mutations were interpreted using the Stanford HIVdb genotyping resistance interpretation algorithm [32].

### 2.1. Statistical Analysis

The analysis included infants with at least 4 viral load (VL) measurements (n = 39) in the first six months after enrolment. The primary outcome was viral load suppression (VLS) defined, for our cohort, as HIV RNA plasma ≤ 400 copies/mL after ART initiation. Although the MOH and WHO cutoff for viral suppression was 1000 copies/mL [33,34] we decided to adopt 400 copies/mL to be able to compare our results with other published studies of the African region. Secondary outcomes were: (1) sustained VLS defined as two or more consecutive measurements of plasma HIV RNA ≤ 400 copies/mL, (2) viral rebound (VR) defined as a measure of VL > 1000 copies/mL after reaching VLS, and (3) HIV transmitted drug resistance. Infants were grouped in VLS and non-VLS. Descriptive analyses included distribution of infants with VLS, sustained VLS and VR, summaries of baseline characteristics of infected infants according to the virologic response, and summaries of the resistance test results. Welch T-test or the non-parametric Wilcoxon and Mann–Whitney tests were used when appropriate to assess the differences among VLS and non-VLS infants. Normality assumption was verified via Shapiro–Wilk test. Categorical features were compared via Fisher’s exact test (two-tailed) or Chi-squared test. Kaplan–Meier estimator [35] was used to calculate the cumulative probability of VLS among all infants who started ART, and according to the pre-ART viral load; cumulative probability of time to rebound was also calculated; Log-rank test was used to compare results among VLS and non-VLS infants [36]. Statistical analysis was performed using Python (version 3.7) [35,37] and *p*-values of <0.05 were considered significant.

### 2.2. Ethical Considerations

The study was approved by the National Bioethics Committee (IRB00002657, reference 102/CNBS/2016) and the Mozambique Ministry of Health (MOH). Caregivers signed an informed consent to participate in the study.

## 3. Results

### 3.1. Characteristics of the Cohort

The median follow up time was 22.5 months (Interquartile Range, IQR 2.20). Infants’ characteristics at baseline are described in Table 1. Median age at diagnosis was 34 days (IQR 12.5), ART initiation was at 34 days (IQR 11.5), median weight was 4.1 Kg (IQR 0.85], weight for height Z-score was ≥−1 SD in 34 (86%) of children. In total, 31 (79.5%) were exclusively breastfeeding, 36 (92.3%) were classified as WHO’s HIV stage I, the median CD4 % was 32% (IQR 13.95), the median CD4 absolute count was 1.956 (IQR 950); the median pre-ART VL was 656,769 copies/mL (IQR 3,198,839) with mean Log_10_ 5.75 (SD 1.10); 21 (51%) infants had a VL ≤ 1,000,000 copies/mL, while 7 (17.9%) had a VL ≥ 6,000,000 copies/mL. A total of 32 (82%) infants received Nevirapine (NVP) post-natal prophylaxis.

Clinical events classified as WHO stage III or IV occurred in infants who did not reach or maintain viral suppression: one child developed toxoplasmosis and died, one had pulmonary tuberculosis, one had encephalopathy and died, and two had severe malnutrition; another infant died after leaving the study at 8 months without reaching VLS. No adverse events were reported during follow up.

### 3.2. Virologic Response to ART

**Figure 1:**
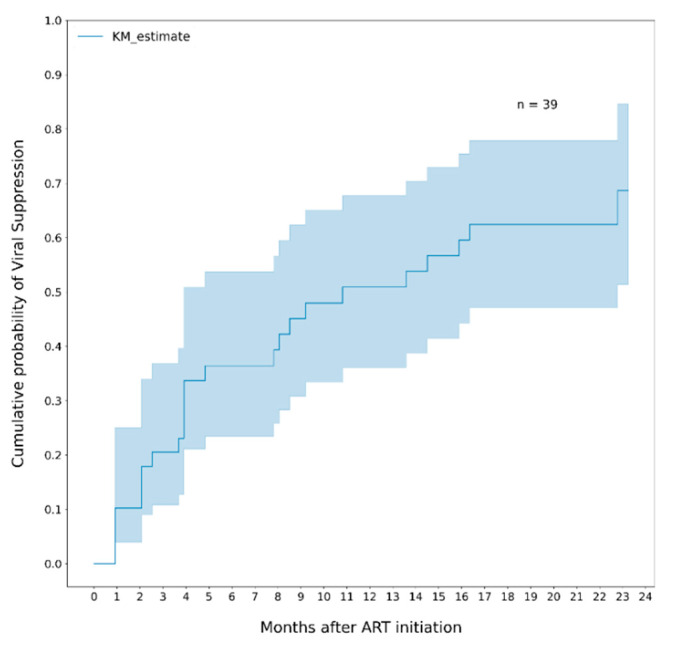
Cumulative probability of viral suppression (VL ≤ 400 copies/mL) among all infants. Note. The area in blue represents the 95% CI.

Twenty-four infants out of 39 (61.5%) reached VLS of plasma HIV RNA ≤ 400 copies/mL after a median time of 3.9 months (IQR 7.5) on ART; 19/24 (79%) maintained viral suppression in two subsequent measurements; 12/24 (50%) had a rebound after reaching VLS, 6/12 (50%) re-suppressed. Among all infants, the cumulative probability of VLS was 36% (95% CI, 26–57) at 6 months of ART, 51% (95% CI, 39–70) at 12 months, 62% (95% CI, 47–78) at 18 months and 69% (95% CI, 51–85) at 24 months (Figure 1).

**Figure 2:**
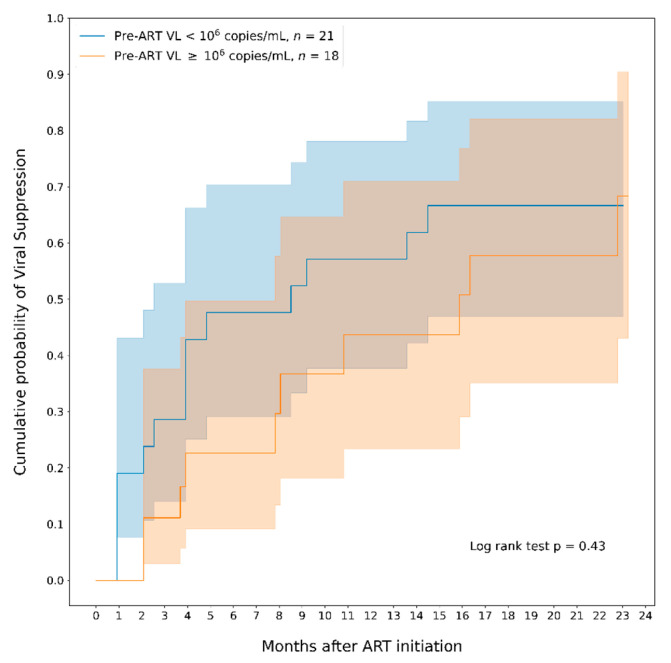
Cumulative probability of viral suppression (VL ≤ 400 copies/mL) among all infants who started ART according to pre-ART viral load. Note. The areas in blue and in orange represent the 95% CI.

We also compared the probability to VLS according to pre-ART viral load using the cut off Log_10_ > 6. Infants with lower pre-ART VL presented a quicker response and had a higher cumulative probability of VLS compared to infants with higher pre-ART VL, especially in the first 6 months after ART initiation, however the difference was not statistically significant (Figure 2).

**Figure 3:**
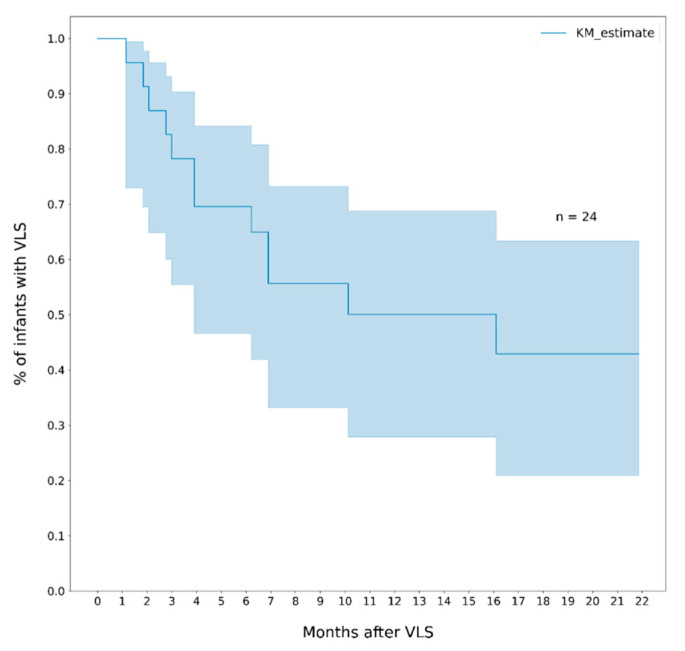
Cumulative probability of viral rebound (VL > 1000 cp/mL) among infants who reached viral suppression (VL ≤ 400 copies/mL). Note. The area in blue represents the 95% CI.

**Figure 4:**
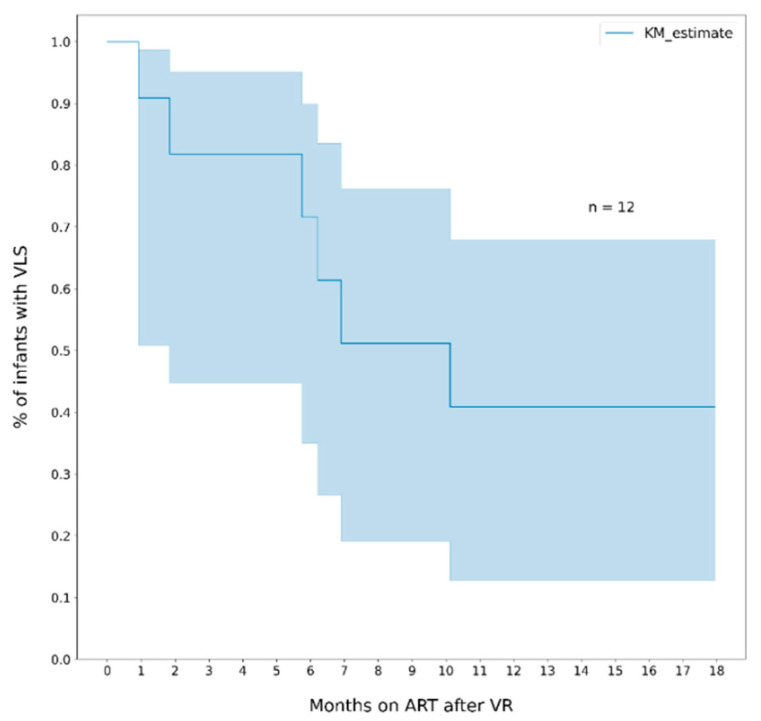
Cumulative probability of re-suppression (VL ≤ 400 copies/mL) among infants who had viral rebound > 1000 copies/mL). Note. The area in blue represents the 95% CI.

The median duration of viral suppression among infants who reached VLS was 7.4 months (IQR 12.6) and among those who rebounded was 3.9 months (IQR 4.3). The cumulative probability of rebound at 6 and 12 months was 30% (95% CI, 16- 53) and 50% (95% CI, 31–72), respectively (Figure 3). The probability of re-suppression among infants who had a rebound was 28% (95% CI, 24–81) within 6 months, and 59% (95% CI, 32–87) within 12 months (Figure 4).

### 3.3. Characteristics of Infants with and without VLS and Their Mothers

The main caregiver was the mother in 37/39 (95%) infants, 2 mothers died soon after delivery and the baby stayed with the grandmother. We compared baseline clinical characteristics of the children with VLS with those without VLS, including few characteristics of the mothers (Table 2). The only significant difference between the two groups was the % CD4 at baseline, VLS infants had higher % CD4 compared to Non VLS infants (*p*-value 0.025). All mothers, except one, were already on ART at the time of delivery, with no significant difference of the duration of treatment (*p*-value 0.77); 7/15 (47%) mothers of non-VLS infants had detectable viremia during follow up, compared to 8/24 (33%) mothers of VLS infants (*p*-value 0.29); 20/24 (83%) mothers of VLS infants compared to 13/15 (87%) mothers of non-VLS infants have disclosed their HIV status to their partner or family (*p*-value 1.00); only 7/39 (18%) mothers had VL test result available at delivery, 6/7 (86%) were not suppressed.

### 3.4. Resistance Test Results

**Figure 5:**
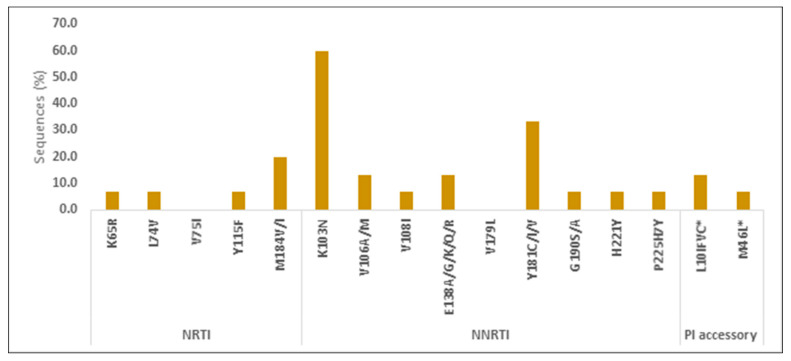
Types and prevalence of pre-ART drug resistance mutations in infants who never reached viral suppression. Note. NRTI: nucleoside reverse transcriptase inhibitor; NNRTI: non-nucleoside reverse transcriptase inhibitor; PI: protease inhibitors.

The resistance test was performed in pre-ART samples of 12/15 (80%) infants who never reached VLS during follow up; 3 infants did not have blood samples stored for this analysis. A total of 2/12 (17%) infants had resistance to ARV used in their regimen: 1 child had high resistance (HR) to 3TC and low resistance (LR) to ABC; 1 child had HR to 3TC and ABC; while 1 child had potential LR to LPV. A total of 11/12 (92%) had resistance to NVP and EFV, these two drugs, however, were not part of the ART regimen, while NVP was used as postnatal prophylaxis per national guidelines. The types and frequencies of resistance mutations are described in Figure 5.

## 4. Discussion

Our analysis is the first describing viral response in a cohort of infants who were started on antiretroviral treatment at one month of age in Mozambique and had repeated measurements of plasma VL during 24-month follow up. Despite being in a study and receiving care from a multidisciplinary specialized team, 62% of infants achieved viral suppression in the two years of follow up: 36% within 6 months and 49% within 12 months after ART initiation. Only 63% of them sustained viral control for 6 months and 42% for 12 months after achieving suppression. The probability to reach viral suppression was 36% at 6 months, 51% at 12 months and 62% at 18 months.

In Mozambique, the national prevalence of viral suppression among 0–14 years old children at the time of the study was 37% [30] and improved to 51% in 2021 [38]. No published data were available for the age group below 2 years of age. Nationally, suboptimal viral suppression was partially due to the large proportion of children still on NVP or EFV based regimens, known to be associated with high primary resistance [39], while transition to the newly approved regimen containing LPV/r or Dolutegravir (DTG) started later. However, infants in our cohort were not on NVP based regimen but on LPV/r, and the results of the resistance test imply that the low suppression rate we found cannot be attributed to primary drug resistance to NVP nor to LPV/r [40,41].

Sub-optimal viral response, between 43% and 63% among children in low and middle-income countries compared to children in high-income countries has been described in a meta-analysis [20]. In South Africa, a cohort of children of 0–12 years old, on a PI-based regimen, presented a cumulative incidence of viral suppression (<1000 copies/mL) at 6, 12 and 24 months of 57.6%, 78.7% and 84.0%, respectively. In the same cohort, infants had a lower viral suppression (<50 copies/mL) rate compared to older children, 46.6% VS 76.9% at 12 months [22], similar to our cohort. In the EPPICC cohort, 62% of infants who started ART below 12 months of age achieved viral suppression after 12 months of treatment, and VLS was less likely to occur in infants younger than 3 months of age compared to those aged 6–12 months [42]. A review of studies describing infants who started ART in the first 6 months of life, found great variability in the initial VLS, from 31% to 72% at 6 months [43,44,45] and from 50% to 72% or 80% at 12 months [13,46,47].

Interestingly, in contrast to past reports [48] the cumulative probability of VLS among infants with higher pre-ART VL did not differ compared to infants with lower pre-ART VL. This finding suggests that viral decay on ART is rapid even in younger infants [21,49].

In line with the literature, we found that despite achieving undetectable viremia, sustaining viral suppression was a challenge. Half of infants who reached VLS had a rapid rebound: 21% of them within 3 months, 29% within 6 months and 46% within 12 months after the first VLS measure. Re-suppression was achieved again in 25% and 50% within 6 and 12 months, respectively after the rebound. Infants with high VL received intensified psychosocial support sessions at the clinic and at home, that can explain the high rate of re-suppression.

Two studies found that the risk of rebound in infants was up to two times higher compared to older children [22,44]. The Ucwaningo cohort, describing viral response in infants starting ART during the neonatal period, showed that only 37% reached and sustained VLS beyond 12 months of age [24]. On the contrary, a study which compared sustained VLS among infants starting ART before 6 months of age with those starting between 6–24 months, found that early ART is protective against viral rebound [43]. Other studies in older children described 38% viral rebound within one year after reaching undetectable plasma viral load [16], while some described lower rate and probability of rebound than that in our cohort [50], but higher in children with opportunistic infections such as tuberculosis and in younger age < 3 years [48,51].

A strength of our study was that in our cohort, it was possible to measure VL at frequent follow up points in time, especially in the first 12 months on ART. These measurements demonstrated the different and unpredictable patterns of viral response among all infants, including those reaching VLS. The low rate of HIV transmitted resistance to ARVs contained in the regimen of a subgroup of infants who never reached VLS and the variability in individual viral response’s trajectories, support the fact that, in our cohort, adherence to ART, played a major role in determining viral response [28,52]. The LPV/r liquid formulation is known for its bitter taste and is poorly accepted by infants and caregivers, and that may have contributed to the poor adherence [53]. In our study, assessment of adherence to treatment relied on caregiver self-report and all caregivers of infants without VLS or with viral rebound reported having some problems in administering the drugs at some point of follow up [54]. They disclosed several dynamic and complex issues in daily routines which affected compliance, which is the main reason of virologic failure also reported elsewhere [16]. In our cohort, we defined good adherence if VL suppression was achieved and poor adherence if it was not achieved or sustained, in the absence of resistance mutation to ARV drug used in the child’s regimen [28].

In Mozambique, viral load test is recommended 6 months after ART initiation and then after 12 months if VLS is achieved, as recommended also by WHO [33,34]. We found that VLS was not sustained during 12 months for the majority of infants as most of them rebounded before the time of the following scheduled test. With the current VL test monitoring schedule, an infant who rebounds within 12 months after suppression will not be promptly identified and will continue with high VL without receiving intense PSS support to address adherence issues that may have emerged in this period. Considering the high rate of viral rebound found in our cohort, we suggest more frequent VL monitoring, at least every 6 months, in children less than 2 years of age after reaching VLS. VL test cost may be an issue for HIV National programs in LMIC, however the number of HIV-infected infants in this age group is small and performing additional VL test outweighs the morbidity and mortality costs that may eventually occur in this population.

Certainly, the PSS component of care remains a key component for mother–baby care irrespective of the availability of the VL test and needs to be strengthened, especially among lay staff who do not have specialized expertise and skills but are trained to provide PSS care to mothers and children in Mozambique. The high rate of rebound calls for urgent action at the programmatic and clinical level to tailor VL monitoring and PSS interventions, especially after the recent roll out of the DTG based regimen. Although resistance test would assist in identifying children who need to switch ARV drugs, this test is not yet affordable and scalable in Mozambique.

Another element we analyzed to better understand the adherence pattern in the mother–baby pairs, was the caregiver’s viral response. Mothers’ viral response during follow up was found to be similar to infant’s viral response in majority of cases: half of mothers of non-VLS infants also had high viral load measurements during follow up compared to a third of mothers of VLS infants, although the difference was not statistically significant. A correlation between child and caregiver’s viral response was previously described in Kenya, where children whose caregivers were not virally suppressed were at higher risk of non-viral suppression [55] and also recently in South Africa for infants starting early ART [24]. The results of VL performed during pregnancy, before enrolment in the study, were not available in the majority of the mothers, as system and laboratory capacity challenges limited optimal VL coverage for all patients living with HIV, including pregnant women and extended the result turn-around-time.

Other factors also played a role in non-VLS infants as the remaining mothers of this group reached suppression themselves, implying that they understood the importance of ART adherence, but somehow were not able to maintain it for their baby. Many studies, although focused on older children, have described barriers to ART adherence in Sub-Saharan African countries and showed that stigma, discrimination and lack of family support are the main barriers reported by caregivers [56,57,58]. In our cohort, the majority of mothers disclosed their status within the family and reported to receive family support, however, that was apparently not enough to ensure full adherence. Partner and family engagement need to be further studied in order to reframe the PSS package of care for mothers and infants living with HIV.

Factors such as the bitter taste of the drug in use (LPV/r), lack of disclosure to partners or family, the resumption of work after delivery and alternative caregivers, are some of the reported challenges that can affect proper adherence [53]. All mothers of non-VLS infants did not miss any visit, thus complying to clinical visits does not guarantee regular administration of ART drugs to their infants.

Majority of mothers in our cohort started ART before or early in pregnancy and supposedly had several opportunities to receive PSS and counselling sessions along the PMTCT care cascade. Despite that, and caregivers having received combined interventions to strengthen adherence to treatment, such as support from mentor mothers [59], the family approach at the clinic and some of the male engagement strategy interventions [60], our findings show that a deeper understanding of factors related to adherence to ART in infants is highly necessary and a different approach is needed for caregivers to mold a strong adherence behavior, engaging family members as well.

These findings highlight the importance of continued focus on mother’s adherence in the post-partum period, and the need to investigate and target any unmet socio-economic needs, as was found in South Africa [24].

The study has several limitations. Firstly, the sample is small. However, results are consistent with data of other similar cohort in Sub Saharan Africa [23,24] and highlight important observational and clinically grounded findings which need further investigation, such as multidimensional correlates of adherence and VLS. The new DTG-based regimen, recently adopted by the country, is expected to improve adherence due the improved formulation, palatability, tolerability and once-daily administration [53,61,62], however VL response should be monitored to preserve this drug from emerging resistance [40,63]. Secondly, the results are generated in a study context which may be different from the ‘real life’ context and may overestimate results such as viral response and retention. However, descriptive results presented are of value as they indicate that if “ideal care conditions” do not guarantee optimal VLS for all infants, then results may be even worse in the “real-life health services scenario” where PSS is provided by lay counsellors and care by non-specialized staff. The strength of the study rests on frequent viral response measurements within two years of treatment, that allowed us to identify the higher probability of rebound in between two standard VL measurements, thereby providing important evidence for possible guidelines review for this age group.

## 5. Conclusions

Low rates of viral suppression and high rates of viral rebound are frequent among HIV-infected infants below two years of age who start ART early and, in our cohort, it is likely attributable to suboptimal adherence to treatment.

An urgent response by the National HIV control program is needed to consider a more frequent viral load monitoring in this age group that will enable prompt identification of children with rebound and timely psychosocial support interventions.

Additionally, a deeper understanding of factors associated with ART adherence in infants and caregivers is fundamental to tailor the PSS model of care to early infancy and to enable caregivers and their families building a strong adherence behavior throughout the continuum of ART care.

## Figures and Tables

**Table 1:** Characteristics of infants included in the analysis at enrollment.

Characteristics at Enrollment	HIV Perinatally Infected Infants n = 39 (%)
**Sex**	
Male	15 (38.46)
Female	24 (61.54)
**Age at HIV diagnosis (days)**	
Median [IQR]	34 [12.5]
**Age at ART initiation (days)**	
Median [IQR]	34 [11.5]
**Time from diagnosis to ART (days)**	
Mean [Min, Max]	1 [0, 14]
**Post-natal prophylaxis, n (%)**	
No	3 (7.69)
Yes	32 (82.05)
Missing	4 (10.26)
**Weight (Kg)**	
Median [IQR]	4.1 [0.85]
**Weight for Height Z -score (WHZ), n (%)**	
≥−1 SD	34 (87.18)
≥2 SD to <−1 SD	5 (12.82)
≥−3 SD to <−2 SD	0 (0)
**Feeding practice at enrollment, n (%)**	
Exclusive breast feeding	31 (79.49)
Mixed feeding	5 (12.82)
Formula feeding	3 (7.69)
**WHO stage, n (%)**	
I	36 (92.31)
II	2 (5.13)
III	0 (0)
IV	1 (2.56)
**CD4 count (cell/mm3)**	
Median [IQR]	1956 [950.25]
**CD4 percentage (%)**	
Median [IQR]	32 [13.95]
**Hemoglobin (g/dl)**	
Median [IQR]	10.6 [2.51]
Missing—n (%)	21 (53.84)
**Pre-ART VL**	
Median (copies/mL) [IQR]	656,769 [3, 198, 839]
Mean log_10_ (SD)	5.75 (1.10)
**VL (copies/mL) n (%)**	
VL ≤ 100,000	5 (12.82)
100,000 < VL ≤ 500,000	12 (30.77)
500,000 < VL ≤ 1,000,000	4 (10.26)
1,000,000 < VL < 6,000,000	11 (28.20)
VL ≥ 6,000,000	7 (17.95)

Note. IQR: interquartile range; ART: antiretroviral treatment; WHO: World Health Organization; SD: standard deviation; VL: viral load.

**Table 2:** Characteristics of infants who reached viral suppression ≤ 400 copies/mL (VLS) and who did not reach viral suppression (Non VLS).

Characteristics of Infants	Virologically Suppressed (VLS)	Non Virologically Suppressed (Non VLS)	*p*-Value
	n = 24	n = 15	
**Sex, n (%)**			
Male	11 (45.83)	4 (26.67)	
Female	13 (54.17)	11 (73.33)	0.32 ^1^
**Age at ART initiation (days)**			
Median [IQR]	33.5 [9.75]	35 [19.00]	0.43 ^2^
**Post-natal prophylaxis, n (%)**			
No	3 (12.5)	0	
Yes	20 (83.33)	12 (80.00)	0.54 ^1^
Missing	1 (4.17)	3 (20.00)	
**WHZ score at baseline, n (%)**			
≥−1 SD	20 (72.22)	14 (93.33)	0.63 ^1^
≥2 SD to <−1 SD	4 (27.78)	1 (6.67)	
≥−3 SD to <−2 SD	0	0	
**WHO stage at baseline, n (%)**			
I	23 (95.83)	13 (86.66)	
II	1 (4.17)	1 (6.67)	0.40 ^4^
III	0	0	
IV	0	1 (6.67)	
**CD4 at baseline, Median [IQR]**			
CD4 (cell/mm3)	2.081 [1184.5]	1.731 [589.50]	0.13 ^3^
CD4 (%)	38.2 [11.3]	26 [12.5]	0.025 ^3^
**Hemoglobin (g/dl) at baseline**			
Median [IQR]	10.6 [2.85]	10.45 [2.27]	0.39 ^3^
Missing (%)	11 (16.67)	10 (66.67)	
**Infant pre-ART VL**			
Median [IQR]	523.995 [3.099.998]	1.462.574 [3.067.486]	0.33 ^2^
Mean log_10_ (SD)	5.54 (1.27)	6.15 (0.66)	
**Mode of delivery, n (%)**			
Vaginal	24 (61.5)	15 (38.5%)	-
Caesarian Section	0	0	
**Mother VL during follow up, n (%)**			
Detectable (≥1000 copies/mL)	8 (33.33)	7 (46.67)	0.29 ^1^
Undetectable (<1000 copies/mL)	14 (58.33)	5 (33.33)	
Missing	2 (8.33)	3 (20.00)	
**Mother ART interruption before delivery, n (%)**			
No	14 (58.33)	6 (40.00)	
Yes	10 (41.67)	7 (46.67)	1.00 ^1^
Missing	0	2 (13.33)	
**Mother time on ART at delivery (days)**			
Median [IQR]	154.0 [268.5]	168 [433.50]	0.77 ^2^
**Mother time on ART at delivery, n (%)**			
<3 months	4 (16.67)	1 (6.67)	0.63 ^4^
3–9 months	11 (45.83)	7 (46.67)	
>9 months	8 (33.33)	5 (33.33)	
Not on ART Missing	1 (4.17) 0	0 2 (13.33)	
**Mother self-disclosure within family, n (%)**			
Yes	20 (83.33)	13 (86.67)	1.00 ^1^
No	4 (16.67)	2 (13.33)	
Missing	0	0	

Note. IQR: interquartile range; WHZ: Weight for Height Z score; SD: standard deviation; WHO: World Health Organization; ART: antiretroviral treatment; VL: viral load. ^1^ Fisher’s Exact Test ^2^ Mann–Whitney ^3^ Welch T-test ^4^ Chi-squared.

## Data Availability

The authors confirm that all data underlying the findings are fully available upon request from the corresponding authors MGL, mglain22@gmail.com and SP, spahwa@med.miami.edu.
